# Impaired coronary microcirculation in type 2 diabetic patients is associated with elevated circulating regulatory T cells and reduced number of IL-21R^+^ T cells

**DOI:** 10.1186/s12933-016-0378-5

**Published:** 2016-04-19

**Authors:** Bernt Johan von Scholten, Alexander Rosendahl, Philip Hasbak, Regine Bergholdt, Andreas Kjaer, Peter Rossing, Tine W. Hansen

**Affiliations:** Department of Diabetic Complications, Steno Diabetes Center, Niels Steensens Vej 1, 2820 Gentofte, Denmark; Diabetes Complications Research, Novo Nordisk A/S, Måløv, Denmark; Department of New Haemophilia, Novo Nordisk A/S, Gentofte, Denmark; Department of Clinical Physiology, Nuclear Medicine & PET and Cluster for Molecular Imaging, Rigshospitalet, Copenhagen, Denmark; University of Copenhagen, Copenhagen, Denmark; Aarhus University Denmark, Aarhus, Denmark; Baxalta Inc, Medical Affairs, Tobaksvej 2, 2860 Søborg, Denmark

**Keywords:** Type 2 diabetes, Flow-cytometry (FACS) analysis, Coronary microcirculation, Coronary flow reserve, Inflammation, Cardiovascular disease, Peripheral blood, Monocyte sub-populations

## Abstract

**Background:**

Low-grade systemic inflammation is considered to participate in the progression of type 2 diabetes (T2D) and in diabetic complications.

**Methods:**

To determine if circulating leukocytes were abnormally regulated in T2D patients, 8-color flow-cytometry (FACS) analysis was performed in a cross-sectional study of 37 T2D patients and 16 controls. Data obtained from the FACS analysis were compared to coronary flow reserve (CFR), assessed by Rb^82^-PET-imaging, to uncover inflammatory signatures associated with impaired CFR.

**Results:**

Presence of T2D was associated with T cell attenuation characterized by reduced overall T cell, Th17, IL-21R^+^, Treg’s and TLR4^+^ T cells, while the monocyte population showed enhanced TLR4 expression. Further, our data revealed reduced M1-like CD11c expression in T2D which was associated with impaired CFR. In contrast, we show, for the first time in T2D, increased TLR4 expression on CD8 T cells, increased Treg cell number and Treg maturation and reduced IL-21R expression on CD8 T cells to be functionally associated with impaired CFR.

**Conclusions:**

Our demonstration that HbA1c inversely correlates to several T cell populations suggests that T cells may play disease modulating roles in T2D. Further, the novel association between impaired CFR and regulatory T cells and IL-21R^+^ T cells imply an intricate balance in maintaining tissue homeostasis in vascular diabetic complications.

**Electronic supplementary material:**

The online version of this article (doi:10.1186/s12933-016-0378-5) contains supplementary material, which is available to authorized users.

## Background

Inflammation is a fundamental biological process not only with the role to enable host protection against pathogens, but also to stimulate and modulate repair and healing when cellular damage occurs. During an inflammatory event, once the initial insult is contained, the primary objective is to restore tissue homeostasis. Failure to appropriately resolve an inflammatory stimulus can result in persistent immune system activation, which can cause tissue damage and disease. Immune cells are the primary effectors of most inflammatory reactions [1]. Evidence suggests that type 2 diabetes (T2D) can be characterized as an inflammatory disease [2]. The vast majority of patients with T2D are overweight to obese, and obesity has been linked to insulin resistance, hyperglycaemia, hyperlipidaemia and elevated inflammatory circulating factors [3]. Elevated levels of acute-phase proteins (e.g. C-reactive protein) as well as several cytokines (e.g. IL-6 and TNFα) are present in patients with T2D and are shown to be modulated during disease progression [4, 5]. Patients with T2D are at high risk of cardiovascular disease [6], and inflammatory processes play an important role in the development of cardiovascular disease [7]. Coronary flow reserve (CFR) is an important physiological parameter in the coronary circulation that reflects the function of large epicardial arteries and the microcirculation. Impaired CFR has previously been described as a powerful, independent correlate to higher cardiac mortality among patients with diabetes [8].

IL-21R positive T cells have been shown to exhibit non-redundant roles in several systemic inflammatory conditions e.g. rheumatoid arthritis [9, 10]. TLR4 is classically associated with myeloid cells and with function as signalling receptor through which the cells sense bacterial infections [11]. Recently, expression and activation of TLR4 on various lymphoid cells, e.g. CD4^+^ T cells, was demonstrated, and has been suggested to act as a negative regulator of the immune response limiting the excessive inflammation [12, 13].

The present study investigated if altered leukocyte sub-populations were present in peripheral blood in patients with T2D compared to healthy controls. Further, the association between leukocyte sub-populations and impaired coronary microcirculation (assessed by CFR) was determined to identify potential novel biomarkers or functional explanations so far not known in T2D subjects.

## Methods

### Study population

From April to December 2013, we conducted a cross-sectional study at Steno Diabetes Center with the aim to assess the value of cardiac positron emission tomography/computed tomography (PET/CT) for non-invasive estimation of microvascular function and structure [14]. A total of 60 consecutive patients with T2D (defined according to the WHO criteria) and 30 non-diabetic controls were included. Participants were aged between 35 and 80 years, with the ability to understand and give informed consent. Participants were excluded if one of the following characteristics were present: (1) history of coronary heart disease or other cardiovascular disease (including stroke) or symptoms from the heart, assessed from patient files and thorough interviews and questionnaires; (2) asthma or chronic obstructive pulmonary disease requiring treatment; (3) kidney disease other than diabetic nephropathy; (4) end-stage renal disease; (5) office blood pressure >200/110 mmHg; (6) second or third degree atrioventricular block; or (7) pregnancy or lactating.

In this per-protocol specified sub-study, 37 of the patients with T2D and 16 of the non-diabetic control persons were included. The inclusion of the participants was based on time of the day for the drawing of the fresh blood sample at Steno Diabetes Center, to allow the flow cytometric analyses to be completed on the same day. Hence, only participants with a drawing of the blood performed before 12 p.m. were included.

Power calculation was performed for the primary study [14] and not for this sub-study. Analyses presented in this manuscript were hypothesis generating, and based on existing literature we anticipated that our sample size was sufficient.

The study was conducted in compliance with the Declaration of Helsinki. All participants gave informed written consent and the study protocol was approved by The Research Ethics Committee, Capital Region of Denmark.

### Clinical measurements

HbA_1c_ was measured by high-performance liquid chromatography and plasma creatinine by an enzymatic method (Hitachi 912, Roche Diagnostics, Germany).

Current smoking was defined as one or more cigarettes/cigars/pipes a day. Weight and height of each participant were measured, and body mass index was calculated as body weight in kilograms divided by height in meter squared.

### Hybrid cardiac positron emission tomography (PET)/computed tomography (CT) imaging

A dynamic, gated cardiac PET study was performed using a hybrid PET/CT scanner in 3D mode (Siemens Biograph™ mCT 128, Siemens, Germany) following administration of 1100 MBq ^82^Rb (Cardiogen∙82^®^, Bracco Diagnostics Inc., USA). Myocardial Blood Flow was calculated automatically with Siemens Syngo MBF 2.3^®^ (Siemens Medical Solutions, USA), using one-compartment tracer kinetic models for ^82^Rb, including regional uptake and clearance parameters, blood to myocardium spill-over and partial volume corrections, and the extraction curve from Lortie et al. [15]. Maximal hyperemia was induced with adenosine infused at 140 μg/kg/min for 6 min. Participants abstained from all caffeine or methylxanthine containing substances for at least 18 h prior to the cardiac PET scan.

### Flow cytometry analysis

Flow cytometric analysis was performed according to standard procedures and samples acquired on a FACSFortessa equipped with blue, red and violet laser followed by data analysis using FACSdiva software (BD Biosciences). Briefly, to inhibit unspecific binding cells were blocked with anti-CD16 (BD Biosciences) and anti-CD32 (BD Pharmingen). This was followed by surface staining of 7-amino-actinomycin D (7-AAD) (Biolegend), CD45 (Biolegend), CD3 (eBioscience), CD4 (Biolegend), CD8 (BD), CD19 (eBioscience) IL-21R (BD Pharmingen), ICOS (eBioscience), CXCR5 (BD), CCR6 (Biolegend), CD14 (BD), CD16 (Biolegend), CD11c (Biolegend), CD163 (Biolegend), TLR4 (Biolegend), CD68 (Biolegend), CD25 (Biolegend), CD127 (BD), CTLA-4 (Bioscience), FoxP3 (Biolegend), GITR (Biolegend).

To determine total cell number in the whole blood samples, total cell count analysis was performed using 123 count beads (eBioscience) according to manufactures description.

Mean fluorescence intensity (MFI) was determined using the geometric mean value after staining with the marker antibody. To verify specificity, a control with a threefold higher concentration of an isotype control antibody was used both in healthy control subjects and in the patients. The MFI after isotype control was similar between healthy and patient samples and hence the value used and shown is the uncorrected MFI value obtained after each marker antibody.

Th17 cells were determined by identifying CD3^+^ CD4^+^ CCR6^+^ cells that upon first 1 h activation with 50 ng/ml PMA and 1 mg/ml ionomycin in (RPMI+ 10 % FCS and P/S, Sigma) and then an additional 5 h in the presence of 10 mg/ml BrefeldinA were positive for IL-17 (eBio64DEC17, eBioscience) after fixation and permeabilization.

### Statistical analysis

Based on their graphical distribution and the results of the Shapiro-Wilk test [(p < 0.05); indicating a non-normal distribution], the skewed variables were log-transformed using the natural (ln) logarithm prior to the t test and linear regression analysis. Differences between patients and controls were assessed by independent samples t test for continuous and χ^2^ for categorical variables.

First, we evaluated the unadjusted levels of the cell markers in the patients and controls.

Next, analysis of covariance compared levels of the cell markers between the patients and controls, and multivariate linear regression analysis was used to assess the independently association with HbA1c and the different cell markers in the total population (n = 53) and CFR in the patients (n = 37). We took the following confounders into account: age, sex, body mass index and smoking. Due to bias of indication, we did not include variables for medical treatment, blood pressure or cholesterol. The adjusted analyses of CFR additionally included diabetes duration. A 2-tailed p < 0.05 was considered statistical significant. Statistical analyses were performed using SAS software (version 9.3; SAS Institute, NC, USA).

## Results

### Cohort description

The total cohort (n = 53) included 67.9 % male and mean (±SD) age was 62.0 ± 9.3 years. Characteristics of participants in the two groups are shown in Table 1. Age and sex distribution did not differ significant between the groups (p ≥ 0.58). However, the patients had higher body mass index compared to the controls (p < 0.001).

**Table 1:** Clinical characteristic of the participants

	Controls (n = 16)	Patients (n = 37)	P
Female, n (%)	6 (38)	11 (30)	0.58
Age (years)	61.4 ± 9.7	62.4 ± 9.4	0.74
Diabetes duration (years)	–	13.2 [8.0–23.0]	–
Body mass index (kg/m^2^)	25.7 ± 3.0	31.7 ± 4.6	<0.001
HbA1c (mmol/mol)	36.5 ± 1.5	55.1 ± 11.9	<0.001
Smokers, n (%)	1 (6.3)	11 (30)	0.06

Data represent percentage (%) or mean ± SD. P values denote differences between controls and patients and were assessed by t test. Diabetes duration was non-normal distributed and is represented as mean [IQR]

The cohort (n = 53) was similar to the total population in the main study (n = 90) in relation to age (p = 0.96), sex (p = 0.50), body mass index (p = 0.20) and smoking (p = 0.54).

### The number of Th17 lymphocytes is reduced, while the TfH cells are increased in T2D patients

**Fig. 1:**
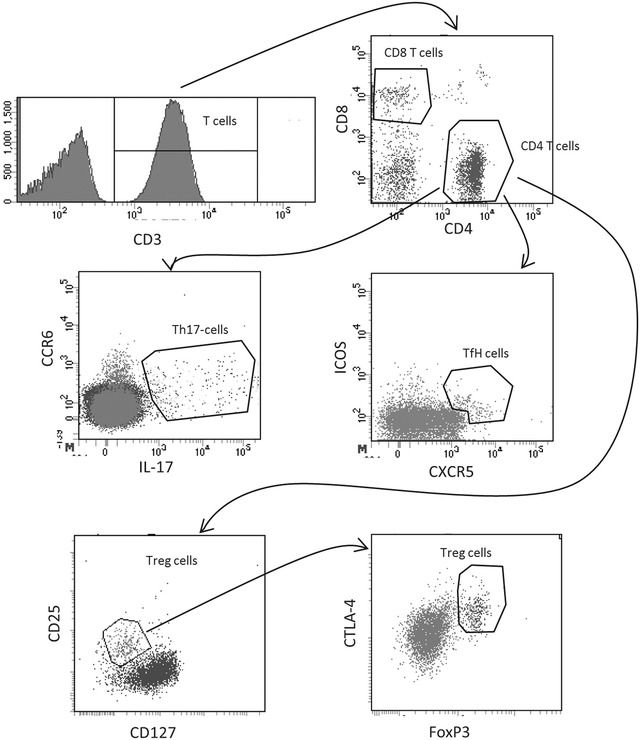
Circulating lymphocyte populations in diabetic patients and healthy controls. A representative gating strategy for T cell subsets is shown after first identifying the cells using gating strategy in Additional file 1: Figure S1. A total of 2 ml blood was analysed and the total number of each cell population was calculated as described in the “Methods” section

The total number of circulating whole blood cells is known to be increased in obese subjects [16]. Detailed analysis in T2D patients is yet to be carefully evaluated and linked to risk parameters. Hence, we determined if the number of T and B cells was modulated in our T2D patient cohort using the gating strategy shown in Additional file 1: Figure S1 and Fig. 1.

**Fig. 2:**
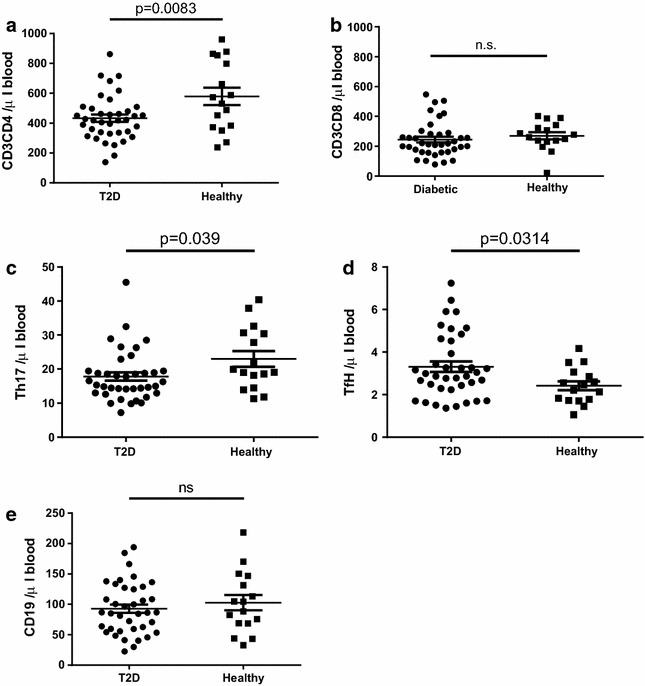
Circulating lymphocyte populations in diabetic patients and healthy controls. The number of CD4 T cells (**a**), CD8 T cells (**b**), Th17 T cells (**c**), TfH T cells (**d**) and B cells (**e**) is shown. A total of 2 ml blood was analysed and the total number of each cell population was calculated as described in the “Methods” section. Each *dot* represents one individual and the *horizontal line* represents the mean value in each group. P values represent difference between groups assessed by t test

The frequency of viable cells in the peripheral blood was similar in healthy and T2D patients determined as frequency of 7AAD exclusion (data not shown). On a cellular level, T2D patients was shown to have significantly (p = 0.008) lower total number of CD4^+^ T cells compared to healthy subjects (433 ± 24 and 579 ± 58 CD4^+^ T cells/µl blood respectively) (Fig. 2a). In contrast, no difference between the T2D patients and healthy subjects was discovered on CD8 T cell level (Fig. 2b). On a CD4 subset level, significant reduction of Th17 cells and a significant increase of TfH cells were observed (Fig. 2c, d). No difference between the T2D patients and healthy subjects was discovered on B cells level (Fig 2e). When adjusting for age, sex, body mass index and smoking the statistical difference between healthy and T2D patients on the CD4 level and the reduction of Th17 cells remained significant (p ≤ 0.003; Table 2).

**Table 2:** Circulating biomarkers in T2D patients vs. controls and in relation to Hba1c as continuous variable in adjusted analyses

Variable	Direction	Controls (n = 16) vs. patients (n = 37)	Hba1c (n = 53)
Circulating lymphocytes			
CD4Tcell_number	−	*0.003*	0.09
CD8tcell_number	+	0.45	0.40
B cells (number) CD19 #Events	−	0.37	0.36
Th17CCR6IL21R_number	−	*0.004*	*0.024*
TfH CXCR5^+^CCR6^+^ICOS^+^ (number)	+	*0.031*	0.76
Circulating monocytes			
Mac_number (CD68)	−	0.45	0.49
M0 Mac number (CD14^+^CD16^−^)	+	0.16	0.74
M1 Mac number (C14dimCD16^+^)	−	*0.006*	0.72
M2 Mac number (CD14^+^CD16+)	−	0.75	0.66
Mac CD11c_MFI (CD68)	−	0.93	0.24
M0 CD14^+^CD16^−^CD11c)	−	0.93	0.20
M1 CD14^dim^CD16^+^CD11c)	+	*0.031*	0.88
M2 CD14^+^CD16^+^CD11c)	−	0.65	0.92
IL-21R on leukocytes in peripheral blood			
CD4Tcell_IL21R_number	−	*<0.0001*	*0.003*
CD8Tcell_IL21R_number	−	*0.0009*	*0.048*
B cell_IL21R_number	−	0.14	0.18
MacIL21R_number	−	*0.003*	0.40
CD4Tcell_IL21R_MFI	−	*<0.0001*	*0.0001*
CD8Tcells_IL21R_MFI	−	*0.004*	*0.006*
B cell IL21R (MFI)	−	*0.023*	0.076
CD68 IL21R APC-A Mean	−	0.38	0.62

Values represent the p values after adjustment for age, sex, body mass index, and smoking. Significant values are highlighted in italics

*MFI* mean fluorescence intensity

+ indicates higher values in patients and with increasing Hba1c; − indicates higher values in controls and with decreasing Hba1c

Taken together, these results reveal that a reduction of the total number of CD4^+^ T cells and of Th17 cells is present in T2D, and that the reduction in this T2D cohort is independent of age, sex, body mass index and smoking.

### Circulating M1-like monocytes are reduced in T2D patients and lower CFR is associated with reduced expression of CD11c^high^ on monocytes

**Fig. 3:**
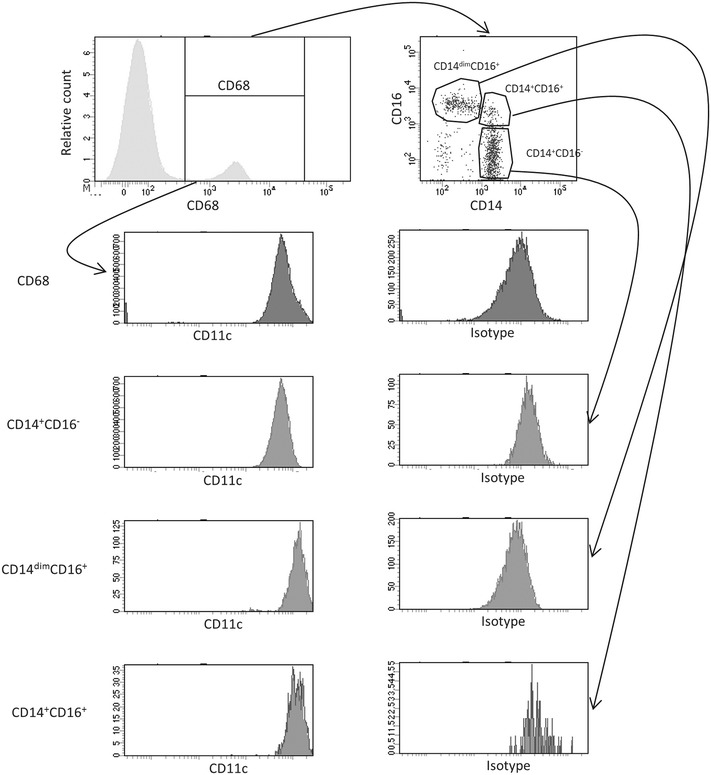
Total number of circulating monocyte populations in diabetic patients and healthy controls. Representative *dot-plot* and *scatter-plots* of CD14 vs CD16 and their expression of CD11c is shown after first identifying the cells using gating strategy in Additional file 1: Figure S1. A total of 2 ml blood was analysed and the total number of each cell population was calculated as described in the “Methods” section

Low grade inflammation is characterized by an enhanced number of M1-like macrophages in adipose tissue and skeletal muscle. The total number of circulating monocytes is not significantly modulated in patients at risk to develop T2D [17], while pre-clinical models of T2D have demonstrated that the monocyte population undergoes a repolarization from an initial M1-like phenotype into a M2-like phenotype in established disease [18]. To address if patients with established T2D display an altered profile of circulating monocyte polarization profile compared to healthy subjects associated with CFR, we performed analysis of peripheral blood in our T2D patient cohort. Using the gating strategy in Additional file 1: Figure S1 and Fig. 3 monocyte subsets were identified.

**Fig. 4:**
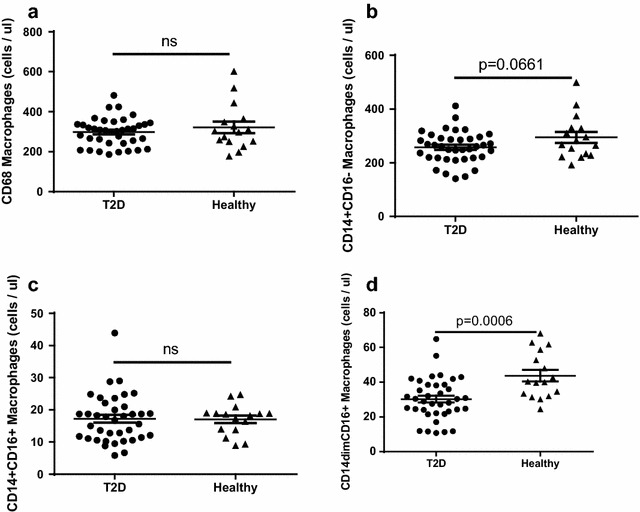
Total number of circulating monocyte populations in diabetic patients and healthy controls. The number of CD68^+^ monocytes (**a**), M0-like CD14^+^CD16^−^ monocytes (**b**), M2-like CD14^+^CD16^+^ monocytes (**c**) and M1-like CD14^dim^CD16^+^ monocytes (**d**) is shown. A total of 2 ml blood was analysed and the total number of each cell population was calculated as described in the “Methods” section. Each *dot* represents one individual and the *horizontal line* represents the mean value in each group. P values represent difference between groups assessed by t test

**Fig. 5:**
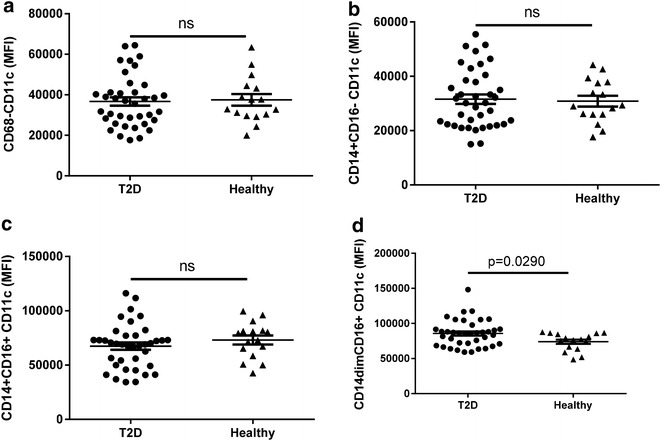
CD11c expression levels on monocyte subpopulations in diabetic patients and healthy controls. The CD11c expression on CD68^+^ monocytes (**a**), M0-like CD14^+^CD16^−^ monocytes (**b**), M2-like CD14^+^CD16^+^ monocytes (**c**) and M1-like CD14^dim^CD16^+^ monocytes (**d**) is shown. A total of 2 ml blood was analysed and the total number of each cell population was calculated as described in the “Methods” section. Each *dot* represents one individual and the *horizontal line* represents the mean value in each group. P values represent difference between groups assessed by t test

Healthy subjects and T2D patients in our cohort both had approximately 300 monocytes/μl blood (Fig. 4a). Analysis of the monocyte compartment using the CD14 and CD16 expression profile as functional markers of M1- and M2-like polarization [19, 20] uncovered a disease specific regulation of the polarization signature (Fig. 4b–d). The undifferentiated CD14^+^CD16^−^ M0-like monocytes, show a moderate reduction in T2D blood compared to healthy subjects (257 ± 9 and 294 ± 20/µl respectively), while the M2-like CD14^+^CD16^+^ cells show no difference between the groups (Fig. 4b, c). Most interestingly, the M1-like CD14^dim^CD16^+^ monocytes, showed a strong and highly significant reduction (p < 0.001) in the T2D patients compared to the healthy subjects (30 ± 2 vs 44 ± 3/µl respectively) (Fig. 4d). No difference between groups was observed after adjusting for age, sex, body mass index, and smoking (p ≥ 0.16; Table 2). In contrast, a significant difference remained between healthy and T2D patients also after adjustment in the M1-like subset of monocytes (p = 0.006; Table 2). To further evaluate the reduction of M1-like monocytes, expression of the M1-associated cell marker CD11c on the monocyte subsets was performed. As expected, no modulation of CD11c expression on the M2- and M0-like monocyte subsets or on the total monocyte population was identified (Fig. 5a–c). In sharp contrast, the remaining CD14^dim^CD16^+^ M1-like monocytes showed a highly significant increase of CD11c surface expression suggesting that although the cell population as such is reduced the remaining cells have augmented capacity to respond in a pro-inflammatory manner (Fig. 5d). When performing adjustment for age, sex, body mass index and smoking, this increase of CD11c surface expression remained significant (p = 0.031; Table 2).

In adjusted models, the CFR was higher with higher surface expression of CD11c both on total CD68^+^ monocytes and on the M2-like CD14^+^CD16^+^ sub-population (p ≤ 0.038; Table 4).

Taken together, the results demonstrate that no significant change is noted on the overall circulating monocyte count in peripheral blood in T2D patients although a small reduction of M1-like monocytes is noted which show an enhanced M1-like polarization. However, most interestingly high expression of the M1-like marker CD11c was correlated to high CFR indicating that M1-like monocyte may be associated with improved cardiac status in diabetic patients.

### Circulating IL-21R^+^ T cells and monocytes are reduced in T2D patients while high CFR is associated with high numbers of IL-21R^+^ CD8 T cells

**Fig. 6:**
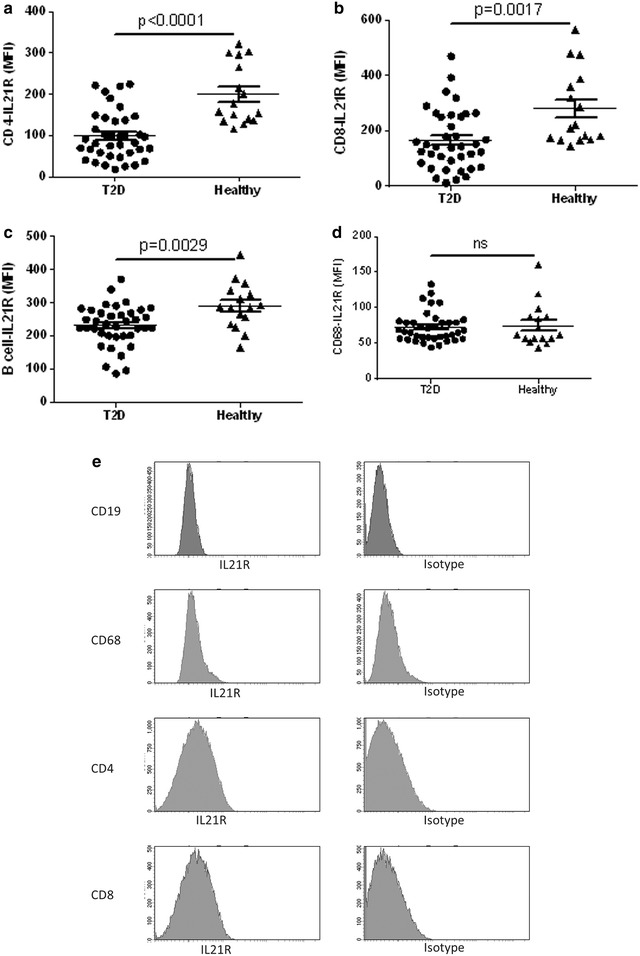
IL-21R expression level on leukocyte populations in diabetic patients and healthy controls. The IL-21R expression on CD4^+^ T cells (**a**), CD8^+^ T cells (**b**), B cells (**c**) and CD68^+^ monocytes (**d**) is shown. Representative histogram analysis of IL-21R expression on CD19^+^, CD68^+^, CD4^+^ and CD8^+^ cells compared to the isotype antibody signal (**e**) that were first identified using the gating strategy from Figs. 1, 2 and Additional file 1: Figure S1. A total of 2 ml blood was analysed and the total number of each cell population was calculated as described in the “Methods” section. Each *dot* represents one individual and the *horizontal line* represents the mean value in each group. P values represent difference between groups assessed by t test

Cytokine receptor activation of leukocyte is essential and determines polarization and effector cell function. In obese subjects it has been demonstrated that IL-21 responsive cells are accumulated in adipose tissue where they are hypothesized to contribute to the metabolic disease progression by fuelling the inflammatory pathways [21]. To determine if a similar IL-21R modulation was present in T2D patients we evaluated the IL-21R surface expression levels (Fig. 6e) and the total number of IL21R^+^ cells and correlated that to the CFR.

The expression level of IL-21R on the circulating CD4^+^ T cells was highly significantly reduced (p < 0.0001) compared to healthy controls (Fig. 6a, e). A similar, but less pronounced reduction of IL-21R expression level was demonstrated on CD8^+^ T cells and B cells (p = 0.0017 and p = 0.0029 respectively) (Fig. 6b, c, e). In contrast, monocytes expressed low and non-modulated levels of IL-21R regardless disease (Fig. 6d, e). This reduced IL-21R expression level resulted in a twofold reduction of the number of IL-21R CD4^+^ and CD8^+^ T cells in the T2D patients (Additional file 1: Figure S1A, B). Interestingly, no modulation was demonstrated on the number of IL-21R positive B cells in T2D patients, whereas a moderate reduction of IL-21R monocytes was determined (Additional file 2: Figure S2C, D).

The reduction of IL-21R expression level and total number of IL-21R positive cells in the T2D cohort remained highly significant in the CD4^+^ and CD8^+^ subsets also after adjustment for age, sex, body mass index and smoking (p ≤ 0.004; Table 2). In fact, the reduction of IL-21R level on CD4^+^ and CD8^+^ T cells and the number of CD4^+^-IL-21R positive cells was demonstrated to be a direct continuous variable for increased HbA1c (p ≤ 0.048; Table 2).

The CFR was higher in patients that had a high number of IL-21R expressing CD8^+^ T cells (p = 0.0024; Table 4). No association was noted on IL-21R^+^CD4 T cell or CD68^+^ monocyte level (Table 4).

Taken together, with increasing HbA1c a strong reduction of IL-21R^+^ T cells and IL-21R expression level was evident. Further the positive correlation between high CFR and elevated CD8^+^IL-21R T cells suggests that these IL-21R^+^ T cells may be associated with better cardiac function in diabetes.

### The number of TLR4 lymphocytes is reduced in T2D patients, but inversely correlated with high CFR

**Fig. 7:**
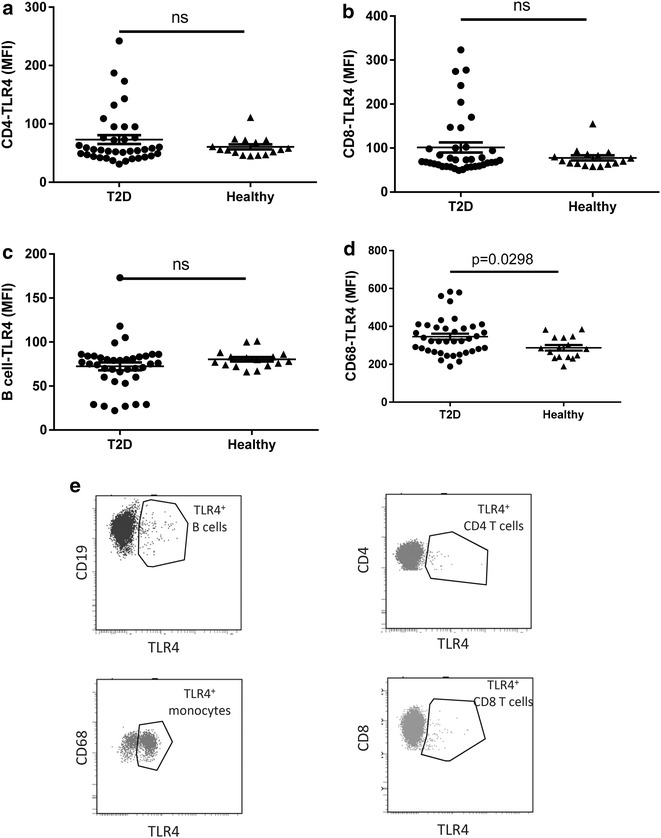
TLR4 expression level on leukocyte populations in diabetic patients and healthy controls. The TLR4 expression on CD4^+^ T cells (**a**), CD8^+^ T cells (**b**), B cells (**c**) and CD68^+^ monocytes (**d**) is shown. Representative* dot plot* analysis of TLR4 expression on CD19, CD68, CD4 and CD8^+^ cells (**e**) that were first identified using the gating strategy from Figs. 1, 2 and Additional file 1: Figure S1. A total of 2 ml blood was analysed and the total number of each cell population was calculated as described in the “Methods” section. Each *dot* represents one individual and the *horizontal line* represents the mean value in each group. P values represent difference between groups assessed by t test

TLR4 expression on B cells and T cells has been associated with immunosuppressive effect [22, 23]. Further, TLR4 expressing B cells are associated with increasing body mass index and an inverse correlation exists between the number of TLR4 T cells and fasting plasma glucose in pre-diabetic as risk [24]. To determine if TLR4 expression level is modulated in patients with T2D and associated with CFR, we performed flow cytometric analysis of the blood in our T2D cohort using the gating strategy from Additional file 1: Figure S1 and Fig. 7e.

The expression level of TLR4 on CD4^+^ and CD8^+^ T cell was not significantly modulated as a population (Fig. 7a, b). However, in both the CD4^+^ and in the CD8^+^ population, six individuals showed highly elevated expression of TLR4 compared to the remaining population in the T2D cohort which was not observed in the healthy subjects (Fig. 7a, b). No significant change in the TLR4 expression on B cells was demonstrated (Fig. 7c). However, the TLR4 expression level on monocytes in the blood was significantly increased in T2D patients compared to healthy subjects (Fig. 7d). In sharp contrast to the expression level of TLR4, the total number of circulating TLR4^+^ CD4^+^, CD8^+^ T cells and B cells was significantly reduced in the T2D cohort (Additional file 3: Figure S3A–C). The circulating monocytes expressing TLR4 was however not increased (Additional file 3: Figure S3D).

In fact, the reduced number of TLR4 CD4^+^ and CD8^+^ T cells remained even after adjustment for age, sex, body mass index and smoking (p ≤ 0.016; Table 3).

**Table 3:** Circulating biomarkers in T2D patients vs. controls and in relation to Hba1c as continuous variable in adjusted analyses

Variable	Direction	Controls (n = 16) vs. patients (n = 37)	Hba1c (n = 53)
TLR4 on leukocytes in peripheral blood			
CD4 T cell TLR4 (MFI)	−	0.064	0.85
CD8^+^ T cell TLR4 (MFI)	−	0.17	0.87
B cell TLR4 (MFI)	−	*0.023*	0.076
Mac TLR4 (MFI)	+	*0.017*	0.58
CD4cell_TLR4_number	−	*<0.0001*	*0.006*
CD8Tcell__TLR4_number	−	*0.016*	*0.019*
BcellsTLR4_number	−	*0.0005*	0.075
MacTLR4_number	+	0.23	0.90
Regulatory T cells and expression of FoxP3 in T cells			
Treg CD25^+^CD127^−^ (number)	−	*0.002*	*0.016*
Treg CD25^+^CD127^−^FoxP3 (number)	−	*0.039*	0.16
Treg CD25^+^CD127^−^GITR^+^ (number)	−	*0.005*	*0.012*
TregCD25CD127FoxP3_MFI	−	0.29	0.076

Values represent the p-values after adjustment for age, sex, body mass index, and smoking. Significant values are highlighted in italics

*MFI* Mean fluorescence intensity

+ indicates higher values in patients and with increasing Hba1c; − indicates higher values in controls and with decreasing Hba1c

The CFR was higher with reduced surface expression of TLR4 on CD8^+^ T cells (p = 0.037; Table 4). There was no correlation between CFR and TLR4 expression on CD4^+^ T cells or on monocytes (Table 4).

**Table 4:** Circulating biomarkers in T2D patients in relation to coronary flow reserve in adjusted analyses

Variable	Direction	Coronary flow reserve (n = 37)
Circulating monocytes		
Mac_number (CD68)	+	0.23
M0 Mac number (CD14^+^ CD16^−^)	+	0.75
M1 Mac number (C14^dim^CD16^+^)	+	0.42
M2 Mac number (CD14^+^CD16^+^)	+	0.32
Mac CD11c_MFI (CD68)	+	*0.038*
M0 CD14^+^CD16^−^CD11c)	+	0.066
M1 CD14^dim^CD16^+^CD11c)	+	0.13
M2 CD14^+^CD16^+^CD11c)	+	*0.037*
IL-21R on leukocytes in peripheral blood		
CD4Tcell_IL21R_number	+	0.23
CD8Tcell_IL21R_number	+	*0.002*
B cell_IL21R_number	+	0.72
MacIL21R_number	−	0.59
CD4Tcell_IL21R_MFI	+	0.093
CD8Tcells_IL21R_MFI	+	0.41
B cell IL21R (MFI)	+	0.83
CD68 IL21R APC-A mean	−	0.68
TLR4 on leukocytes in peripheral blood		
CD4 T cell TLR4 (MFI)	+	0.17
CD8^+^ T cell TLR4 (MFI)	−	*0.037*
B cell TLR4 (MFI)	−	0.60
Mac TLR4 (MFI)	+	0.75
CD4cell_TLR4_number	+	0.97
CD8Tcell__TLR4_number	−	0.32
BcellsTLR4_number	+	0.22
MacTLR4_number	+	0.36
Regulatory T cells and expression of FoxP3 in T cells		
Treg CD25^+^CD127^−^ (number)	−	0.88
Treg CD25^+^CD127^−^FoxP3 (number)	−	*0.029*
Treg CD25^+^CD127^−^GITR + (number)	−	0.31
TregCD25CD127FoxP3_MFI	−	*0.001*

Values represent the p values after adjustment for age, sex, body mass index, smoking and diabetes duration. Significant values are highlighted in italics

*MFI* Mean fluorescence intensity

+ indicates higher values with increasing coronary flow reserve; − indicates lower values with increasing coronary flow reserve

Taken together, although individual subjects showed enhanced expression level of TLR4 on the T cells, the number of TLR4^+^ -T cells and B cells in the T2D cohort was significantly decreased. Further, the data demonstrate that the reduced expression of TLR4 on T cells was correlated with enhanced CFR suggesting that TLR4 lymphocytes may be related to cardiac functions in diabetes.

### Attenuated presence of regulatory T cells in T2D patients and inverse association between Treg presence and CFR

**Fig. 8:**
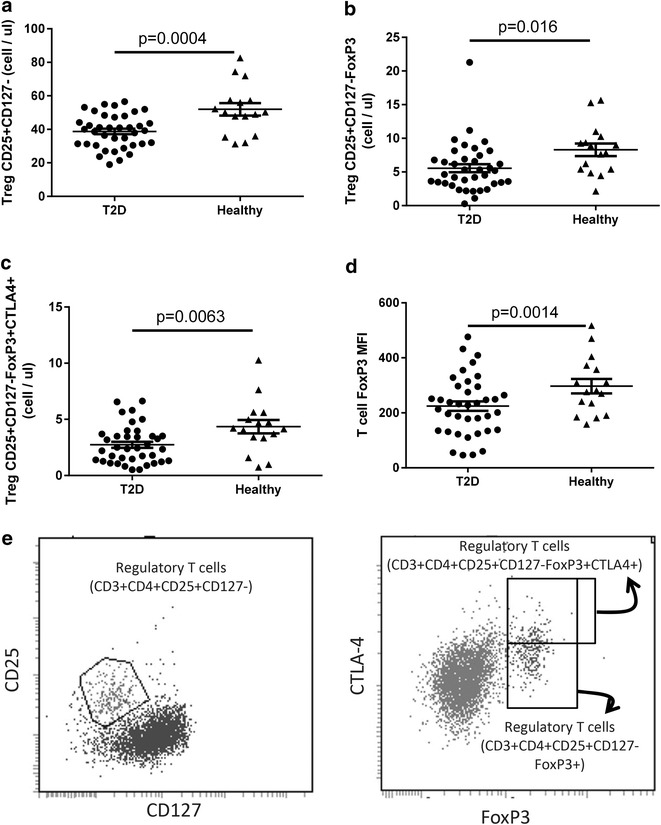
Circulating regulatory populations in diabetic patients and healthy controls. The number of Tregs (CD4^+^CD25^+^CD127^−^) T cells (**a**), FoxP3^high^ Tregs (CD4^+^CD25^+^ CD127^−^) T cells (**b**), CTLA4^high^ Tregs (CD4^+^CD25^+^ CD127^−^FoxP3^high^) T cells (**c**) and FoxP3 MFI on Tregs (CD4^+^CD25^+^ CD127^−^) T cells (**d**). Representative dot plot analysis of Treg sub-populations (CD3^+^CD4^+^CD25^+^CD127^−^; CD3^+^CD4^+^CD25^+^CD127^−^FoxP3^+^; CD3^+^CD4^+^CD25^+^CD127^−^FoxP3^+^CTLA4^+^) (**e**) that were first identified using the gating strategy from Figs. 1, 2 and Additional file 1: Figure S1. A total of 2 ml blood was analysed and the total number of each cell population was calculated as described in the “Methods” section. Each *dot* represents one individual and the *horizontal line* represents the mean value in each group. P values represent difference between groups assessed by t test. *MFI* mean fluorescence intensity

Aberrant inflammation is present in several autoimmune diseases with enhanced activity in e.g. rheumatoid arthritis as well as reduced activity in certain tumours [25]. Recently this was demonstrated to relate to a reduced presence and activity of regulatory lymphocytes in autoimmune diseases and inversely an augmented activity of regulatory cells in tumours. To determine if regulatory T cells were modulated in our T2D cohort and associated with CFR, flow cytometric analysis of various Treg sub-populations in the peripheral blood was performed using gating strategy from Figs. 1 and 8e.

The mean number of regulatory T cells (CD3^+^CD4^+^CD25^high^CD127^neg^) phenotype was 52 ± 4 cells per µl blood in healthy controls (Fig. 8a). A highly significant 26 ± 3 % reduction (p < 0.001) of the number of CD25^high^CD127^neg^ Tregs was present in the T2D patients (Fig. 8a). While on average 8.3 ± 0.9 cells/µl blood co-expressed the regulatory transcription factor FoxP3 in healthy subjects, a significant (p = 0.016) 33 % reduction to 5.6 ± 0.4 cells/µl blood was evident in T2D patients (Fig. 8b). Further detailed analysis of the FoxP3^+^ cells revealed that 4.4 ± 0.4 cells/µl blood in healthy subjects also co-expressing CTLA4 while only 2.7 ± 0.3 cells/µl (p = 0.006; 38 % reduction) were CTLT4^+^ in T2D patients (Fig. 8c). To evaluate if the Tregs present in the blood had different transcriptional machinery associated with regulatory functions in healthy and diabetic subjects, the expression level of the transcription factor FoxP3 was determined. The results clearly demonstrate that T cells in healthy subjects have a significantly higher expression level of FoxP3 than in T2D subjects in our cohort (Fig. 8d).

When adjusted for age, sex, body mass index and smoking the results remained significant (p ≤ 0.039; Table 3), except for the expression level of FoxP3 on the CD25^+^CD127^−^ cells (p = 0.29; Table 3).

Intriguingly and in sharp contrast, in adjusted analyses, the CFR was higher with reduced number of regulatory T cells (p = 0.029; Table 4). This inverse correlation was even more pronounced with highly significantly lower expression of FoxP3 in the regulatory T cells in patients with high CFR (p = 0.001; Table 4).

Taken together, this demonstrates that the number of regulatory cells is highly significantly reduced in T2D patients and that the remaining T cells show an attenuated differentiation towards a regulatory phenotype. Most importantly, high CFR was associated with low presence of regulatory T cells, suggesting that regulatory T cells might be linked to progression of cardiac complications in patients with T2D.

## Discussion

In the present study we show that presence of diabetes is associated with a general T cell attenuation characterized by reduced overall T cell, Th17, IL-21R^+^, Treg’s and TLR4^+^ T cell count, while the monocyte population shows enhanced TLR4 expression. Further, our data revealed a reduced M1-like CD11c expression on our T2D cohort which was functionally associated with lower CFR. In contrast, we show for the first time in a T2D cohort increased TLR4 expression on CD8 T cells, increased Treg cell number and Treg maturation and reduced IL-21R expression on CD8 T cells to be functionally associated with impaired CFR. Even with early and multifactorial treatment, T2D patients show an enhanced incidence of cardiovascular complications. Presence of low grade systemic and local tissue inflammation is now a well-established characteristic and strong evidence exists linking vascular diabetic complications to inflammatory pathways [26]. However, to the best of our knowledge, we are the first to evaluate the relation between systemic inflammatory signature and CFR in T2D patients. These findings might provide important novel information potentially guiding future therapeutic approaches to reduce the burden of cardiovascular complications in T2D patients.

Signalling through the IL-21 receptor promotes proliferation and contributes to effector mechanisms like viral elimination and antibody switch [27]. Interestingly, our T2D cohort demonstrated a significantly reduced expression level of IL-21R on circulating T cells compared to healthy subjects. Fabrizi et al. recently demonstrated an increase of IL-21R mRNA transcripts in adipose tissue in obese compared to healthy subjects [21]. Through local regulation of IRF4, these adipose immigrating IL-21R^+^ T cells performed immunoregulation which reduced the activity of Treg cells in the adipose tissue fuelling the low grade inflammation [21]. These IL-21R^+^ T cells migrated to the obesity adipose tissue through specific chemokine patterns not identified in the study by Fabrizi et al. [21]. A similar tissue homing of IL-21R T cells in diabetic patients has previously been shown to be dependent on the activity of CCR9 for gut-homing [28]. Due to both the gut-homing (CCR9 dependent) and adipose tissue homing (undefined) the net-result in the systemic compartment will be a reciprocal decrease of IL-21R^+^ circulating cells which is in accordance with and strengthen our novel observations. Furthermore, we recently demonstrated in a cohort of 20 subjects at risk to develop diabetes an inverse correlation of the frequency of IL-21R^+^ T cells with increased body mass index [17]. Thus, our novel findings in established T2D extend our previous observations in patients at risk of developing T2D that the systemic presence of IL-21R^+^ CD4 T cells is reduced in obesity, pre-diabetes and T2D. Based on the Fabrizi and the McGuire data this may be a direct consequence of altered migratory patterns [21, 28]. Hence, circulating IL-21R^+^ T cells might act as a novel biomarker allowing identification of patients with metabolic syndromes.

Interestingly, on a CD4 subset level, a significant increase of TfH cells was observed in T2D patients with HbA1c. As these cells migrate through CXCR5, the accumulation in the systemic compartment may indicate absence of or reduced levels of local chemokine production of CXCL13. Interaction with CXCR5/CXCL13 is essential for the organization and establishment of lymphoid tissues [29]. In human visceral leishmaniasis infections reduce lymphoid tissue production of CXCL13 results in impaired B/T cell interaction leading to sub-optimal adoptive immunity [30]. Hence it is tempting to speculate that the reduced numbers of circulating T cells in our cohort may be associated with a similar mechanism.

Patients with a reduced number of systemic IL-21R^+^ CD8 T cells presented with a significantly lower CFR, indicative of impaired coronary microcirculation and hence a worse cardiovascular prognosis. This phenomenon was not observed on CD4^+^ T cell level in the cohort. Discrepancies in tissue accumulation have previously been described in viral pre-clinical models and in human HIV patients [31, 32]. With the distinct expression profile of IL-21R^+^ on CD4 and CD8 cells it is intriguing to speculate that IL-21R^+^ effector CD8 T cells rather than contributing to disease progression delivers yet to be identified signals protecting the vasculature in T2D. IL-21 and IL-21R inhibition has been frequently debated in the literature, where both higher and lower levels have been linked to atherosclerosis [33]. Our novel finding with impaired CFR in patients with a reduced number of circulating CD8-IL21R^+^ T cells add additional important knowledge currently lacking in the understanding of the cardiovascular complications associated with T2D potentially aiding in the contradictory data around IL-21 in vasculature complications. Consequently, low IL-21R on circulating CD8 T cells may be considered to be a good biomarker to identify patients with increased risk of having impaired CFR.

M1 macrophages have been linked to low-grade inflammation, insulin resistance and weight gain [34]. Tissue recruited monocytes differentiate into an M1 macrophage phenotype and their accumulation leads to an imbalance between M1 and M2 macrophages locally in the tissue [34]. Increased pro-inflammatory cytokine production from M1 macrophages and/or reduced anti-inflammatory signals from the M2 macrophages promote adipose tissue dysfunction and impairs glucose tolerance in the early stages of diabetes [1]. In contrast, in late-stage established diabetes the macrophages exhibit a polarization profile closely associated with remodelling M2-like macrophages in mice [18, 20]. This disease-dependent maturation and polarization of the inflammatory response may directly contribute to the excessive tissue fibrosis which is the hallmark of diabetic complications [18, 35]. Herein we now extend our previous pre-clinical observations and demonstrate that macrophages associated with pro-inflammation (M1-like) are significantly reduced in circulation also in clinical samples from T2D patients. The pro-inflammatory M1-like cells may have accumulated in adipose and skeletal muscle contributing to the insulin resistance resulting in a reciprocal loss in the systemic compartment [36]. The observation that the remaining peripheral monocytes in patients with reduced CFR show lower expression of CD11c, i.e. classical M1-like monocytes, is intriguing. These cells are associated with production of pro-inflammatory cytokines but not with repair and remodelling mechanisms [37]. Further, CD11c on monocytes has been shown essential for monocyte adherence and migration into atherosclerotic plaques in pre-clinical models [38]. The discrepancies between the pre-clinical macrovascular diseases and the clinical microvascular disorder could be due to analyses in different disease segments and at different locations. With our demonstration that the overall M1-like monocytes are reduced in number, but the remaining monocytes express elevated CD11c indicates that low CD11c expression could be used as a functional biomarker to identify patients with impaired CFR.

Regulatory T cells control adaptive immune responses by suppressing T cells, NK cells, NKT cells, B cells and dendritic cells [39]. Regulatory T cells and subsets of CD4^+^ Th2 cells are known to secrete anti-inflammatory and homeostatic cytokines with the ability to inhibit and modulate macrophage recruitment and activation state [40]. Previous studies have shown that in obese adipose tissue, the regulatory T cells were diminished leaving these patients at risk to progress into diabetes [41]. In line with this, we demonstrate that the number of circulating regulatory cells is reduced in patients with T2D compared to healthy controls. Further, the remaining T cells showed an attenuated differentiation towards a regulatory phenotype. Similar reductions have been described in other chronic diseases often associated with immune deficiency (e.g. HIV) where it is strongly associated with a poor prognosis and active progression of disease development [42].

Transforming growth factor beta (TGF-β) is a crucial pleiotropic cytokine associated with the development of Tregs and Th17 cells [43]. In our cohort of patients with T2D, we showed a reduction of both Tregs and Th17. This suggests that neither the pro-inflammatory Th17 nor the regulatory Tregs may be suitable functional biomarkers of T2D as they are both down-regulated in the same disease stage. In sharp contrast, they may be good disease and functional biomarkers for coronary diabetic complications as they both are strongly associated with impaired coronary microcirculation. Most interestingly, enhanced numbers of Tregs as well as the elevated expression level of FoxP3 on the regulatory T cells was associated with impaired coronary microcirculation in our cohort. TGF-β promotes tissue remodelling and is highly associated with the development of Tregs [44]. In healthy subjects this is in balance; accordingly, injury induces a pro-inflammatory response which after a short period is turned down by locally produced TGF-β that promotes Tregs leading to repair [44]. In contrast, in patients with chronic disease excessive TGF-β is produced in the “termination phase”. This leads to polarization towards M2-like macrophages, tissue remodelling, stiffening of tissues and in the end of this cascade to atherosclerosis [45]. In our T2D cohort, patients with increased Tregs had a lower CFR, indicative of impaired coronary microcirculation. Our data might suggest that augmented down-regulation is associated with excessive locally produced TGF-β leading to stiffening of the tissue. Hence, the balance is disturbed which may favour remodelling rather than normal tissue repair.

Along the same line, TLR4 expression on CD8 T cells was lower in patients with reduced CFR. This is highly interesting as TLR4 expressing lymphocytes has been described as regulators of the immune responses by providing signals that terminate pro-inflammation, while TLR4 expressing macrophages classically is known as potentiators of pro-inflammation [46]. The detrimental role of TLR4 in atherosclerosis through monocytes and macrophages is well established [47]. Our novel finding that reduced TLR4 on CD8 lymphocytes correlates to an impaired coronary microcirculation in T2D patients is a novel and potentially important finding placing immune-modulating CD8 T cells in a novel tissue protecting role. Thus, caution should be taken when considering therapies inhibiting TLR4 in vascular diabetic complications as this might risk to further worsen the coronary microcirculation by inhibiting the TLR4^+^CD8 T cells.

Matrougui et al. [48] elegantly showed that injections with Tregs in hypotensive mice lead to reduced macrophage activation and infiltration, lower TNFa levels and improved coronary arteriolar endothelium-dependent relaxation. Hence while Tregs may play a protective role in the development of coronary arteriolar endothelial dysfunction in hypertension, our data suggest that in chronic diabetic induced disease increased Tregs may actually correlate to impaired coronary microcirculation.

Recent findings also depict the role of inflammation and immune dysregulation on coronary microvascular function. A study, including patients with non-obstructive coronary artery disease, demonstrated an independent relationship between higher levels of soluble urokinase-type plasminogen activator receptor (suPAR), a surrogate of systemic inflammation and immune function, and lower CFR [49].

Multiple factors can be involved in impairment of CFR including hyperglycemia, insulin resistance, endothelial dysfunction and increased cardiac sympathetic activity [50]. Moreover, recent cross sectional studies have shown lower CFR to be associated with decreased aortic distensibility [51] and left ventricular diastolic dysfunction [52] in asymptomatic patients with type 2 diabetes. Treatment of diabetic hyperglycemia may improve CFR [50]. However, in a short-term (10 weeks) study, the GLP-1 analogue liraglutide did not have any significant effect on CFR (assessed by Doppler flow echocardiography) in patients with type 2 diabetes [53]. These conflicting results might be explained by short observation times and selection criteria.

### Strengths and limitations

A major strength of this study is the use and combination of two advanced procedures; FACS analyses and assessment of CFR by cardiac Rb^82^ PET/CT (a sensitive marker of coronary microcirculation (currently considered the gold standard) [50] in a clinical relevant setting including both T2D patients and healthy controls. Despite these strengths, limitations merit considerations. As the aim of this study was hypothesis generating and thought provoking, we investigated a long series of markers. Further, due to the demanding procedures applied, the number of included participants is relatively low leading to limited power.

## Conclusions

Herein we describe for the first time novel insights linking inflammatory signatures to the CFR. A pattern emerged suggesting low presence of Tregs, FoxP3 expression and TLR4^+^CD8 T cells together with enhanced of IL21R^+^ CD8 T cells and CD11c M1-like monocytes in peripheral blood to be associated with high CFR. Our data imply that low CFR seems to be associated with an attenuated inflammatory signature in combination with elevated regulatory activity. Our data provide important clues about inflammation-diabetes-CFR that may be useful when designing novel therapeutic agents filling the current unmet medical need as well as providing novel non-invasive functional biomarkers useful to identify patients at risk to develop severe T2D complications.

## Additional files

10.1186/s12933-016-0378-5 Gating strategy to identify living singlet leukocytes.
10.1186/s12933-016-0378-5 Total number of IL-21R^+^ leukocyte populations in diabetic patients and healthy controls. The number of IL-21R^+^ CD4^+^ T cells (A), CD8^+^ T cells (B), B cells (C) and CD68^+^ monocytes (D) is shown. A total of 2 ml blood was analysed and the total number of each cell population was calculated as described in the Materials and Methods section. Each dot represents one individual and the horizontal line represents the mean value in each group. P values represent difference between groups assessed by t test.
10.1186/s12933-016-0378-5 Total number of TLR4^+^ leukocyte populations in diabetic patients and healthy controls. The number of TLR4^+^ CD4^+^ T cells (A), CD8^+^ T cells (B), B cells (C) and CD68^+^ monocytes (D) is shown. A total of 2 ml blood was analysed and the total number of each cell population was calculated as described in the Materials and Methods section. Each dot represents one individual and the horizontal line represents the mean value in each group. P values represent difference between groups assessed by t test.
