# Supplementary figures and images for: Impaired coronary microcirculation in type 2 diabetic patients is associated with elevated circulating regulatory T cells and reduced number of IL-21R+ T cells
Source: Cardiovasc Diabetol. 2016 Apr 19;15:67. doi: 10.1186/s12933-016-0378-5 (PMC4837587; doi:10.1186/s12933-016-0378-5)

## Slide 1
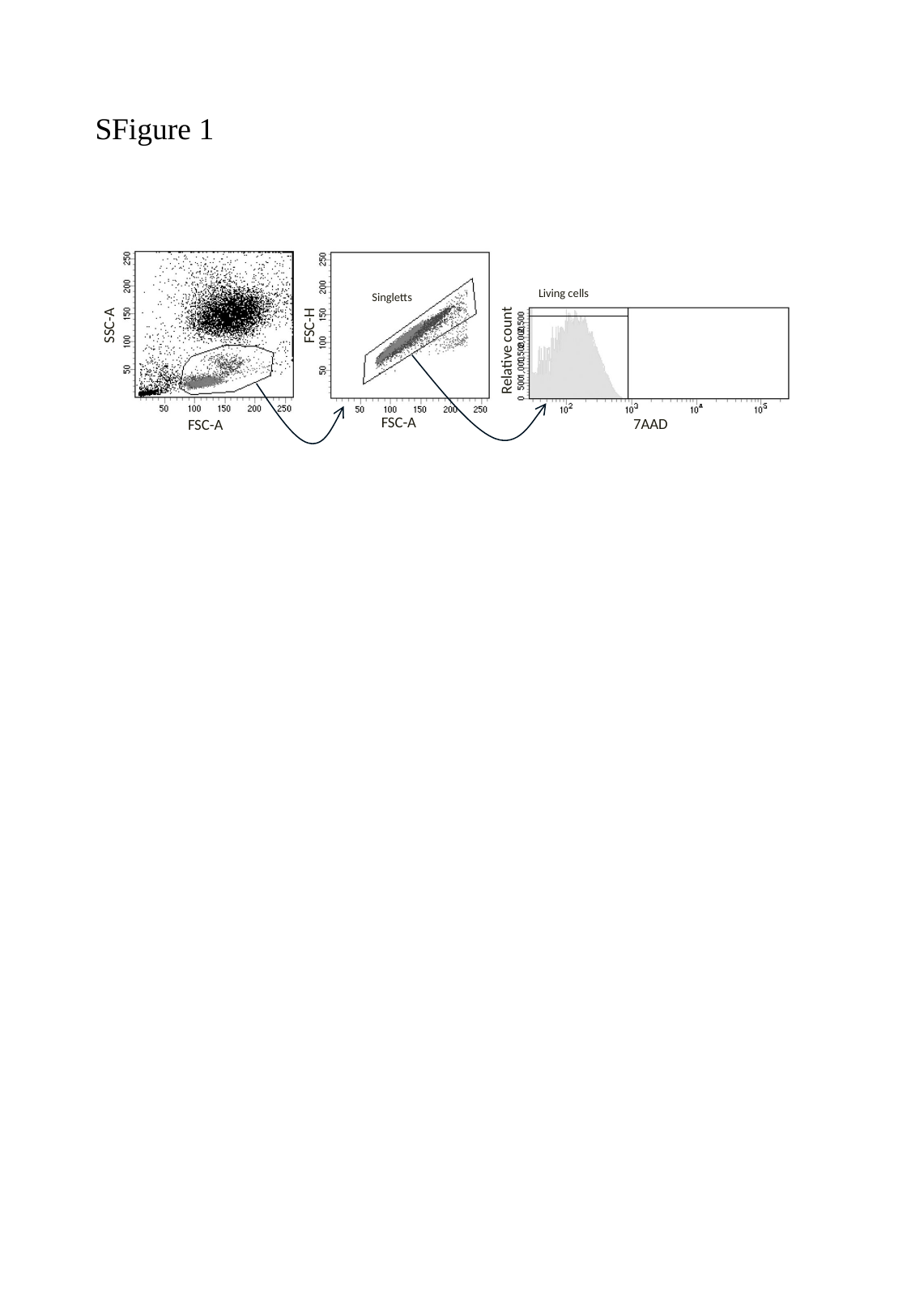

SFigure 1
Living cells
Singletts
SSC-A
FSC-H
Relative count
FSC-A
7AAD
FSC-A

Supplement: Supplementary file 1 — 10.1186/s12933-016-0378-5 Gating strategy to identify living singlet leukocytes. [file 12933_2016_378_MOESM1_ESM.pptx]

## Slide 1
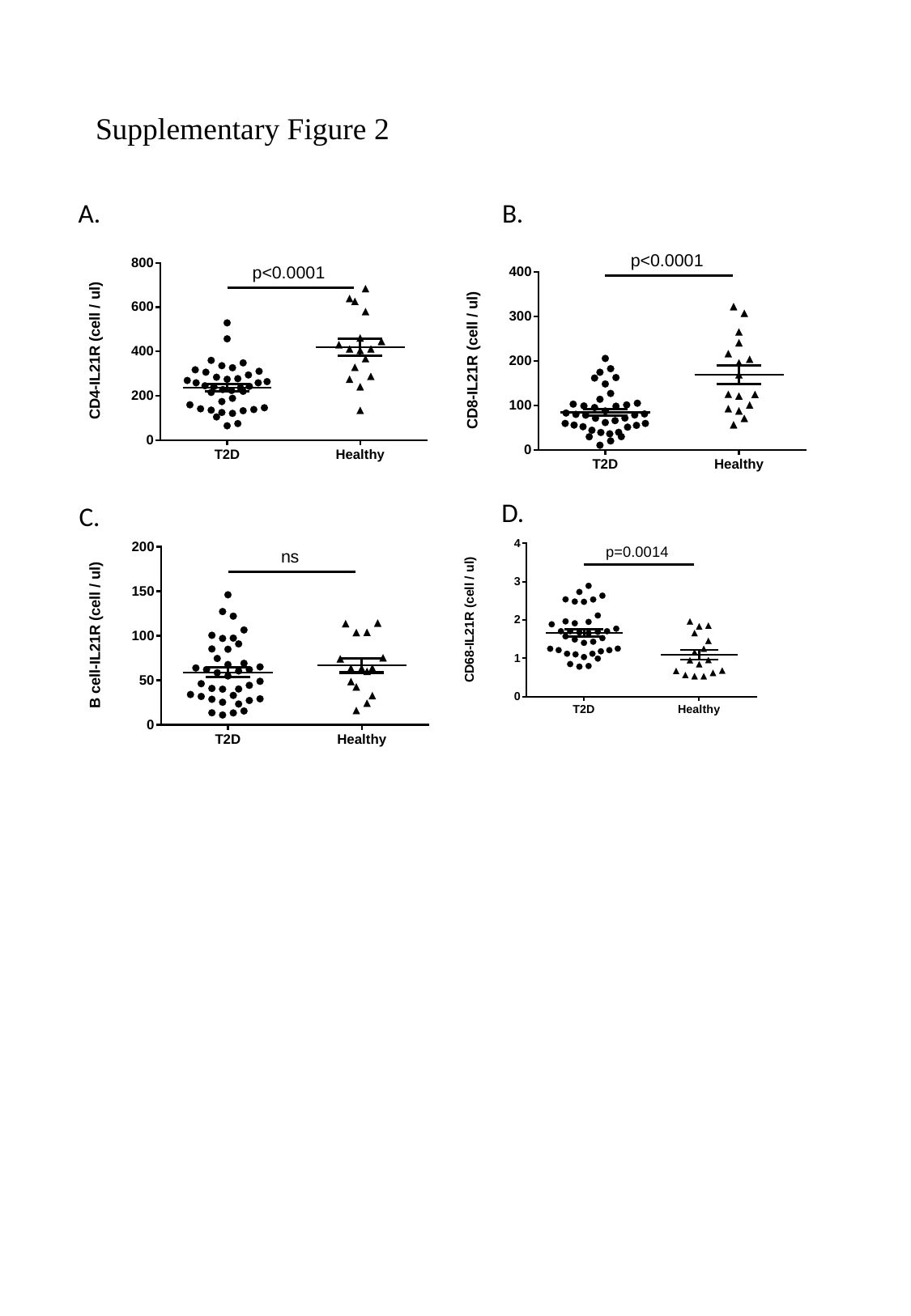

Supplementary Figure 2
A.
B.
D.
C.

Supplement: Supplementary file 2 — 10.1186/s12933-016-0378-5 Total number of IL-21R+ leukocyte populations in diabetic patients and healthy controls. The number of IL-21R+ CD4+ T cells (A), CD8+ T cells (B), B cells (C) and CD68+ monocytes (D) is shown. A total of 2 ml blood was analysed and the total number of each cell population was calculated as described in the Materials and Methods section. Each dot represents one individual and the horizontal line represents the mean value in each group. P values represent difference between groups assessed by t test. [file 12933_2016_378_MOESM2_ESM.pptx]

## Slide 1
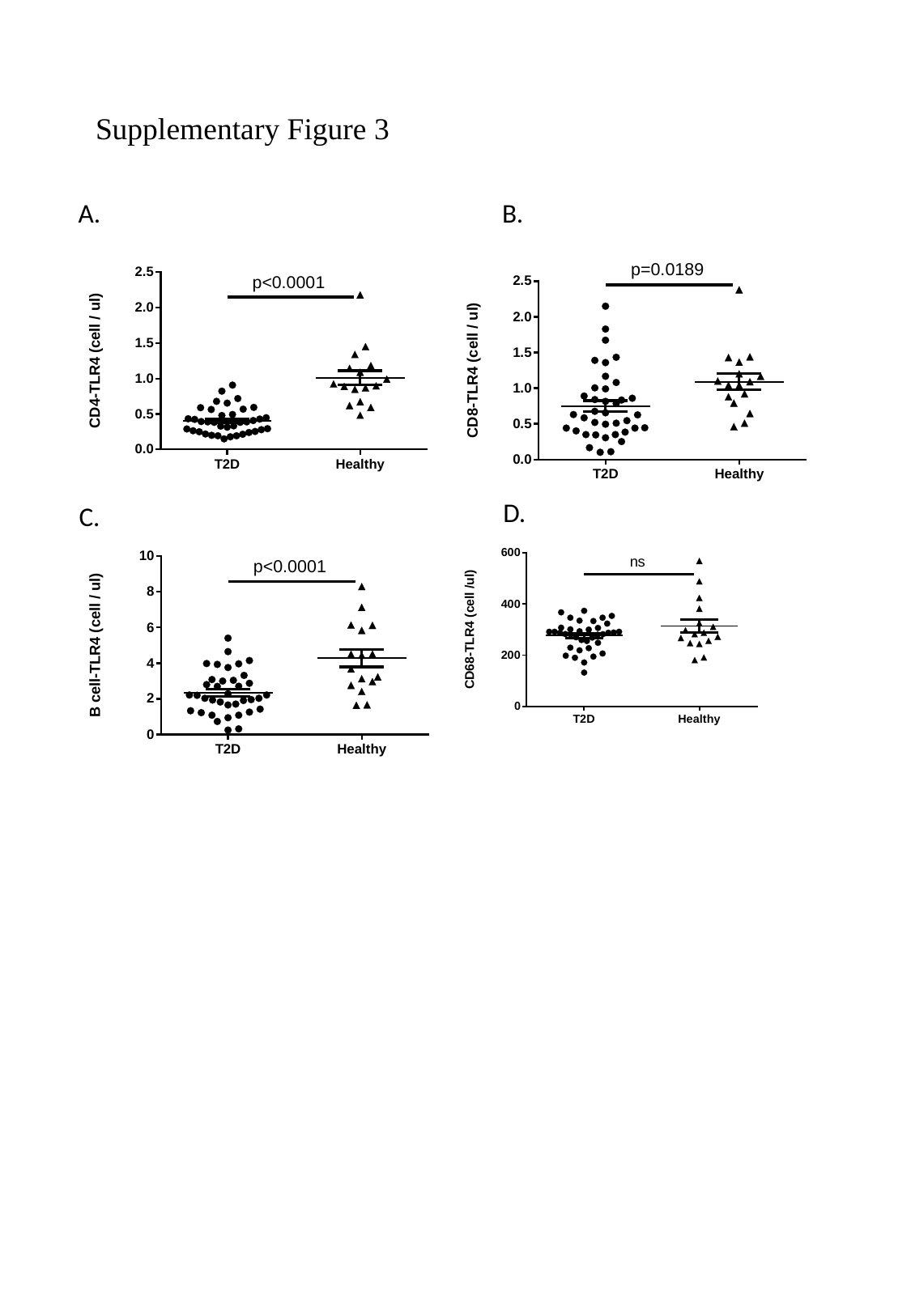

Supplementary Figure 3
A.
B.
D.
C.

Supplement: Supplementary file 3 — 10.1186/s12933-016-0378-5 Total number of TLR4+ leukocyte populations in diabetic patients and healthy controls. The number of TLR4+ CD4+ T cells (A), CD8+ T cells (B), B cells (C) and CD68+ monocytes (D) is shown. A total of 2 ml blood was analysed and the total number of each cell population was calculated as described in the Materials and Methods section. Each dot represents one individual and the horizontal line represents the mean value in each group. P values represent difference between groups assessed by t test. [file 12933_2016_378_MOESM3_ESM.pptx]
